# Intestinal Microbiota Influence Doxorubicin Responsiveness in Triple-Negative Breast Cancer

**DOI:** 10.3390/cancers14194849

**Published:** 2022-10-04

**Authors:** Alaa Bawaneh, Adam S. Wilson, Nicole Levi, Marissa M. Howard-McNatt, Akiko Chiba, David R. Soto-Pantoja, Katherine L. Cook

**Affiliations:** 1 Department of Surgery, Wake Forest School of Medicine, Winston-Salem, NC 27157, USA; 2 Department of Integrative Physiology and Pharmacology, Wake Forest University School of Medicine, Winston-Salem, NC 27157, USA; 3 Department of Plastic and Reconstructive Surgery, Wake Forest University School of Medicine, Winston-Salem, NC 27157, USA; 4 Department of Surgery, Duke University Medical Center, Durham, NC 27710, USA; 5 Department of Cancer Biology, Wake Forest University School of Medicine, Winston-Salem, NC 27157, USA; 6 Comprehensive Cancer Center, Wake Forest University School of Medicine, Winston-Salem, NC 27157, USA

**Keywords:** triple-negative breast cancer, microbiome, inflammation, doxorubicin, lipopolysaccharide, metagenomic sequencing, *Akkermansia muciniphila*, proliferation, apoptosis

## Abstract

**Simple Summary:**

Triple-negative breast cancer (TNBC) is a highly aggressive tumor with a poor prognosis and limited treatment options. Finding new approaches to improve TNBC outcomes and response to treatment is critical. Using the 4T1 murine TNBC model, we show the effect of neoadjuvant chemotherapy in modulating gut microbiota contents. In addition, gut microbiota could be used in the future as a predictive biomarker for doxorubicin responsiveness. Modulating gut microbiota through antibiotics, diet-derived fecal microbiota transplantation, or by exogenous LPS administration impact tumor growth, response to treatment, and metastasis formation. Therefore, harnessing gut microbiota contents could be considered a promising approach in affecting triple-negative breast cancer responsiveness to chemotherapy treatment.

**Abstract:**

Triple-negative breast cancer (TNBC) is highly aggressive with a poor 5-year survival rate. Targeted therapy options are limited and most TNBC patients are treated with chemotherapy. This study aimed to determine whether doxorubicin (Dox) shifts the gut microbiome and whether gut microbiome populations influence chemotherapeutic responsiveness. Female BALB/c mice (n = 115) were injected with 4T1-luciferase cells (a murine syngeneic TNBC model) and treated with Dox and/or antibiotics, high-fat diet-derived fecal microbiota transplant (HFD-FMT), or exogenous lipopolysaccharide (LPS). Metagenomic sequencing was performed on fecal DNA samples. Mice that received Dox were stratified into Dox responders or Dox nonresponders. Mice from the Dox responders and antibiotics + Dox groups displayed reduced tumor weight and metastatic burden. Metagenomic analysis showed that Dox was associated with increased *Akkermansia muciniphila* proportional abundance. Moreover, Dox responders showed an elevated proportional abundance of *Akkermansia muciniphila* prior to Dox treatment. HFD-FMT potentiated tumor growth and decreased Dox responsiveness. Indeed, lipopolysaccharide, a structural component of Gram-negative bacteria, was increased in the plasma of Dox nonresponders and FMT + Dox mice. Treatment with exogenous LPS increases intestinal inflammation, reduces Dox responsiveness, and increases lung metastasis. Taken together, we show that modulating the gut microbiota through antibiotics, HFD-FMT, or by administering LPS influenced TNBC chemotherapy responsiveness, lung metastasis, and intestinal inflammation.

## 1. Introduction

In the United States, one out of eight women is at risk of developing breast cancer during their lifetime. Breast cancer is the second leading cause of cancer-related death in women and is characterized by different molecular subtypes according to receptor expression [1]. Triple-negative breast cancer (TNBC) comprises approximately 15–20% of all breast cancer cases [2]. TNBC cells lack estrogen receptor-α (ER) and progesterone receptor (PR) and do not overexpress human epidermal growth factor receptor 2 (HER2) [3]. Accordingly, owing to the lack of expression of these receptors, targeted therapy options for TNBC are limited. Chemotherapy-based regimens are considered the standard of care for improving disease outcomes [4]. Neoadjuvant chemotherapy is a combination of chemotherapeutic agents, often comprising of anthracyclines (doxorubicin), alkylating agents (cyclophosphamide), and taxanes (paclitaxel, docetaxel), administered to patients with breast cancer to reduce tumor size and limit lymph node involvement prior to definitive surgical treatment [5]. The inclusion of anthracycline-based regimens in neoadjuvant settings is still very important [6]. One of the most common anthracycline agents used in the clinic, doxorubicin (Dox), is an anti-tumor chemotherapy routinely used to treat several cancers [7,8]. Dox can induce toxic side effects, including gastrointestinal disturbances and intestinal mucositis, suggesting that Dox may affect the gut microbiome [9].

The microbiome is the community of microorganisms that live in and on an organism and play a critical role in health and disease development [10]. The human body consists of approximately 100 trillion microbial cells [11], and the vast majority of the bacterial microbiome is present in the gastrointestinal tract (mainly the colon), which comprises over 70% of the human microbiota [12]. Moreover, each individual has a distinct gut microbiota composition, which is influenced by many factors, including age, race, diet, exercise, medical conditions, drugs, and antibiotic use. Homeostasis between the host and microbial entities should be maintained for normal body functioning and survival. Gut homeostasis can be achieved through several mechanisms, which are as follows: (1) the eubiotic system produces selective toxins that prevent pathogenic bacterial growth, (2) maintaining a state of low inflammation in the host; and (3) physical separation between the mucus layer and epithelial cells, thus limiting the interaction between the microbiome and the immune system [13]. Any disruption of this balance results in dysbiosis, a state of barrier failure, inflammation, generation of cytokines/chemokines, and ROS formation, which may promote the progression of diseases such as inflammatory bowel disease [14], obesity [15], and breast cancer [16].

A comprehensive metagenomic comparison of the gut microbiota in breast cancer patients and healthy controls found no significant taxonomic differences in the gut between premenopausal breast cancer patients and controls. In contrast, several bacterial species were found to be enriched in postmenopausal patients relative to the controls: *Escherichia coli*, *Klebsiella sp*_1_1_55, *Prevotella amnii*, *Enterococcus gallinarum*, *Actinomyces* sp. HPA0247, *Shewanella putrefaciens*, and *Erwinia amylovora.* In contrast, various species were less abundant in postmenopausal patients, including *Eubacterium eligens* and *Lactobacillus vaginalis*, suggesting an association between gut microbiota and the development of postmenopausal breast cancer. However, it is essential to note that a potential limitation of this study may be the confounding effect of menopause on the gut microbiome, as depletion of circulating estrogens was associated with shifts in gut microbiome populations that were not specifically controlled for in the healthy control population [17]. Another study investigated the diversity and composition of the gut microbiota by fecal 16 S-rRNA sequencing in postmenopausal women newly diagnosed with breast cancer and found that postmenopausal women’s fecal microbiota showed low diversity, different composition, and metabolic pathways compared to healthy control women. This suggests that disruption of gut microbiota homeostasis may be associated with breast cancer development and highlights menopause status as an important factor in determining gut microbiota composition [18]. Furthermore, a recent study showed that disturbances in the gut microbiome could promote breast cancer metastasis in a mouse model of hormone receptor-positive breast cancer, where the authors investigated the effect of antibiotic-induced dysbiosis on tumor development and metastasis formation. The study showed that inducing gut dysbiosis in an animal model had no significant effect on the growth of the primary tumor; however, it increased the dissemination of tumor cells into the blood, lymph nodes, and lungs, suggesting gut dysbiosis as a regulator of metastases [19]. Taken together, these studies highlight the potential role of the gut microbiome in mediating breast cancer risk through several mechanisms, including estrogen metabolism, bacterial metabolites, short-chain fatty acid production, immune modulation, and microbial translocation [20]. Moreover, oral and systemic drugs are able to modulate the gut microbiome, suggesting that cancer therapy may result in shifting microbiota populations to affect outcomes. Cyclophosphamide (CTX) can alter small intestine microbiota composition in mice bearing subcutaneous tumors and induce a selective translocation of distinct Gram-positive bacterial species of the Firmicutes phyla *Lactobacilli* and *Enterococci* into secondary lymphoid tissues, such as mesenteric lymph nodes and spleens. In addition, germ-free mice and mice treated with antibiotics to selectively ablate Gram-positive bacteria showed compromised anti-tumor efficacy of CTX, suggesting that microbiota may facilitate CTX therapeutic efficacy [21]. A previous study revealed that CpG-oligonucleotide immunotherapy and oxaliplatin (platinum chemotherapeutic) efficacy were attenuated in antibiotic-treated and germ-free mice due to lower cytokine production and reduced ROS generation after treatment [22]. Other studies showed that Dox is transformed into other metabolites via specific strains of gut bacteria. *Raoultella planticola* is considered a potent Dox inactivator. Under anaerobic conditions, bacteria can deglycosylate doxorubicin into 7-deoxydoxorubicinol and 7-deoxydoxorubicinolone; this process may result in a reduction in doxorubicin efficacy and toxicity [23].

Outside of the gut, the breast tissue has its own microbiota with a distinct population compared with the skin and other organs [24,25]. Interestingly the presence of tumors, breast tumor sub-type, and tumor grade influence microbial profiles [16,26,27]. These microbial populations have a role in both benign and malignant diseases. For instance, it has been shown that the microbial contents of benign tumors are more similar to healthy tissues than those of malignant tumors [16]. Dysbiotic breast microbiota and microbial-associated molecular patterns (MAMPs) influence genetic instability, DNA damage and modulate inflammatory and immune system responses, leading to malignant progression [28,29]. Previous work by our group demonstrated the effect of neoadjuvant chemotherapy on breast tumor microbiota; 16S sequencing of breast tumors showed a reduction in diversity of microbiota in breast tumor tissues of patients who received neoadjuvant chemotherapy compared to tumors from treatment-naïve patients [30]. Moreover, the study showed neoadjuvant chemotherapy increased tumoral *Pseudomonas* abundance and decreased *Streptococcus* populations. The development of distant metastasis correlated with increased primary tumor abundance of *Brevundimonas* and *Staphylococcus*, suggesting that the composition of intra-tumoral microbiota could be modulated by therapy to impact drug responsiveness and metastatic progression [30]. Overall, several studies highlighted the role of tumor microbiota contents’ role in breast cancer development, metastatic progression, and response to treatment [31].

The purpose of this study was to determine whether Dox modifies the gut microbiome and whether, in turn, the gut microbiome influences Dox chemotherapy responsiveness in TNBC. Using a syngeneic TNBC murine model treated with broad-spectrum antibiotics to sterilize the gut microbiome, high-fat diet-derived fecal microbiota transplant (FMT), or lipopolysaccharide (LPS) injections to model metabolic endotoxemia/leaky gut parameters, we investigated Dox chemotherapeutic responsiveness, intestinal inflammation, and metastatic development. Furthermore, we associated certain gut taxa with chemotherapy outcomes by metagenomic sequencing of fecal DNA. We showed that administration of systemic Dox modifies the gut microbiome and modulating the gut microbiota through high-fat diet-FMT reduced Dox efficacy, highlighting the importance of the gut microbiome composition in breast cancer development and response to therapy.

## 2. Materials and Methods

### 2.1. In Vivo TNBC Tumor Murine Model

Female 7-week-old BALB/c mice (n = 45) were purchased from Jackson Laboratory and injected with 1 × 10^6^ 4T1-luciferase tagged cells (TNBC) in the left fourth mammary gland fat pad to induce tumors. Mice were divided into the following subgroups: untreated control group, doxorubicin-treated group, antibiotic-treated group, and doxorubicin + antibiotics. The antibiotic cocktail was a mixture of ampicillin (1 mg/mL), colistin (1 mg/mL), and streptomycin (5 mg/mL) administered in drinking water and replaced weekly [21]. Once the tumor volume reached 100 mm^3^, 2.5 mg/kg of IV-doxorubicin was injected into the tail veins 1× weekly for 4 weeks to reach an accumulated dose of 10 mg/kg. Fecal samples were collected before treatment (T0) and after the 4th dose of doxorubicin (T4). Tumor volumes were measured every three days using calipers. IVIS imaging (In Vivo Imaging System^®^, PerkinElmer; Waltham, MA, USA) was performed weekly to monitor tumor growth and metastatic lesions in the distal organs. At the study endpoint, mice were humanely euthanized using approved methods; plasma, tumors, lungs, and intestines were harvested for analysis. All protocols were approved by the Animal Care and Use Committee of the Wake Forest School of Medicine (protocols A18-020 and A21-047), and all procedures were performed in accordance with relevant guidelines and regulations.

### 2.2. Fecal Microbiota Transplantation (FMT) Animal Model

Fresh fecal samples were collected aseptically from high-fat diet-fed mice (Envigo Teklad diet; catalog# TD.06414) and mixed with sterile saline at a 1 g:2 mL ratio. The samples were vortexed until the fecal pellets were homogenously suspended, centrifuged to remove any remaining large particulates, and the supernatant was transferred to a new sterile tube. Female BALB/c mice (n = 40) were fed a control diet (Envigo Teklad diet; catalog number TD.08806). Mice were divided into the following three groups: control group, dox-treated group administered saline gavage, and high-fat diet fecal microbiota transplant (FMT) + Dox. The FMT gavage was administered at 100 µL twice weekly over 3 weeks. After the establishment of the gut microbiota, 1 × 10^6^ 4T1-luc cells were injected into the left fourth mammary gland fat pad to induce tumor growth. Tumor-bearing animals were then treated according to group designation.

### 2.3. Lipopolysaccharide (LPS) Study Animal Model

Female 7-week-old BALB/c mice (n = 30) were injected with 1 × 10^6^ 4T1-luc cells into the left fourth mammary gland fat pad. To induce systemic inflammation, 5 mg/kg lipopolysaccharide (LPS) (Sigma-Aldrich, catalog #L2630) was intraperitoneally injected once a week for the duration of the study [32]. Mice were divided into control, LPS-only, and Dox + LPS groups. Once the tumors reached 100 mm^3^, the mice were treated according to the group designation.

### 2.4. IVIS Imaging

In vivo bioluminescent imaging was performed for the tracking, monitoring, and quantification of signals, which were controlled by the acquisition and analysis software Living Image^®^. Luciferase (100 mg/kg) was administered intraperitoneally (i. p.). Mice were then anesthetized (1–3% isoflurane) for 15 min before image acquisition, and luminescence was captured using a ventral view in the in vivo imaging system (IVIS^®^ Imaging System). This process was performed weekly to monitor tumor growth and distant metastasis.

### 2.5. Immunohistochemistry

Tumors, lungs, and intestines were fixed in 4% paraformaldehyde for 24 h before embedding in paraffin. Embedded lung and intestinal tissues were cut into 5 µm thick sections and stained with hematoxylin and eosin (H&E). H&E staining of the intestines showed inflammation by measuring villi length and muscularis thickness. Lung metastatic lesions were quantified, and if lesions were detected, the lesion area was measured using ImageJ software. Cross-sectional paraffin-embedded intestinal tissue was stained for goblet cells using Alcian blue (pH 2.5) (Abcam Cat#, ab150662) staining protocol. Tumor sections were stained with Ki-67 antibody (Cell Signaling Cat#, 12202;1:100 dilution), F4/80 antibody (Cell Signaling Cat#, 70076; 1:500 dilution), and cleaved caspase 3 antibody (Cell Signaling Cat #9661; 1:400 dilution) using the Dako Envision Plus IHC staining kit and visualized using DAB chromogen to investigate tumor proliferation, inflammation, and apoptosis among the groups. Staining was visualized using the Mantra Quantitative Pathology Image System with 10×, 20×, and 40× objectives; four representative images from each tissue type were quantified and averaged.

### 2.6. Study Approval and Breast Cancer Plasma Procurement

This study was approved by our institutional review board (IRB00045734) in accordance with HHS regulations for the protection of human research subjects. Subjects who were female and diagnosed with invasive ductal carcinoma were retrospectively identified as those in the Sentinel Lymph Node Mapping and Surgical Outcomes (IRB00008131) database. For inclusion in the study, subjects must have provided written consent for the Advanced Tumor/Tissue Bank (BG04-104) and plasma for research in the Tumor Bank. Patient demographics, preoperative variables, surgical details, and clinical outcomes were recorded. The patient characteristics are described in Supplementary Table S1.

### 2.7. Enzyme-Linked Immunosorbent Assay (ELISA)

Plasma was collected from the animals at the end of the study, stored at −80 °C, and analyzed. LPS was measured using an LPS ELISA Kit (LSBio, Cat# LS-F17912) following the manufacturer’s protocol, and the plate was read immediately at 450 nm using a Bio-Rad Benchmark Plus microplate spectrophotometer. Plasma from breast cancer patients was used to measure lipopolysaccharide-binding protein (LBP) using the Human LBP ELISA Kit (Invitrogen, Cat# EH297RB), following the manufacturer’s protocol.

### 2.8. Metagenomics Sequencing

DNA was isolated from 100 mg of frozen feces using the Qiagen DNeasy PowerSoil Pro Kit (Valencia, CA, USA), and metagenomic sequencing was performed by CosmosID, Inc. (Rockville, MD, USA). As previously described [33], DNA libraries were prepared using the Illumina Nextera XT preparation kit (San Diego, CA, USA). Library quantity was assessed using a Qubit Fluorometer (Thermo Fisher Scientific, Wilmington, DE, USA) and sequenced on an Illumina HiSeq platform to generate 150-bp paired-end reads.

### 2.9. Statistical Analysis

Data are presented as mean ± SEM. Statistical differences in most studies were evaluated using Student’s *t*-test (pairwise) or one-way ANOVA, followed by Bonferroni post hoc tests to compare all groups (GraphPad Prism 9 software). Tumor volumes were obtained from measurements of the longest perpendicular axes ((long axes) × (short axes)^2^)/2). Tumor volumes over time were evaluated by a two-way ANOVA, followed by Bonferroni post hoc tests to compare all groups and time points. The criterion for statistical significance was set at *p* < 0.05.

## 3. Results

### 3.1. Antibiotics Administration Impacted TNBC Dox Responsiveness and Lung Metastasis Formation

**Figure 1:**
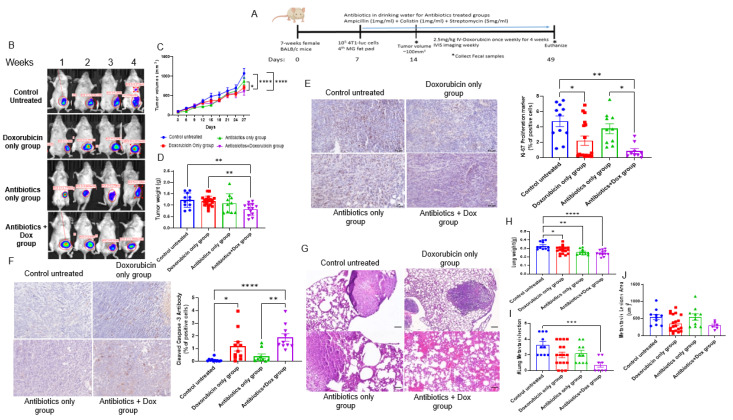
Combination of antibiotics and doxorubicin reduce tumor growth and lung metastasis formation. (**A**) Schematic design of murine TNBC model. Image produced by www.biorender.com. (**B**) Representative images of In Vivo Imaging System (IVIS) of mice after each dose of doxorubicin (Dox) injection over 4-week of treatment. (**C**) Tumor volume was measured every three days and recorded in mm^3^. n = 10–15; * *p* = 0.01, **** *p* < 0.0001. (**D**) Tumor weights in grams. n = 9–15; ** *p* = 0.008. (**E**) Tumor proliferation marker Ki-67 by IHC immunoreactivity. n = 10–15; * *p* = 0.01, ** *p* = 0.001. (**F**) Apoptosis marker (cleaved caspase 3) in tumors by IHC immunoreactivity. n = 10–11; * *p* < 0.05, ** *p* = 0.002, **** *p* < 0.0001. (**G)** Representative images of H&E-stained lungs from each treatment group. Lung weights (**H**), lung lesions (**I**), and metastatic lesion area (**J**) measured in the lung. n = 6–15; scale bar = 100 µm; * *p* = 0.02, ** *p* = 0.001, *** *p* = 0.0009, **** *p* < 0.0001.

Patients with breast cancer who undergo mastectomy or reconstructive surgeries are prescribed prophylactic antibiotics to prevent surgical site infections [34]. However, it is well recognized that antimicrobial administration can dramatically affect the composition and function of the gastrointestinal microbiome [35]. To determine the effect of systemic administration of antibiotics on Dox efficacy in TNBC, we administered a broad-spectrum antibiotic cocktail (ampicillin, colistin, and streptomycin) in the drinking water of mice bearing 4T1 TNBC tumors and administered Dox (Figure 1A). Antibiotic administration had no overall effect on tumor growth. Dox alone or Dox + antibiotics decreased tumor volume (Figure 1B, C). In contrast, Dox + antibiotic-treated animals displayed significantly reduced tumor weight at the end of the study compared to control or Dox-treated animals (Figure 1D). Dox alone or Dox + antibiotic administration reduced tumor proliferation, as determined by Ki67 immunoreactivity (Figure 1E), and increased apoptosis, as determined by cleaved caspase 3 immunoreactivity (Figure 1F), suggesting that antibiotics did not negatively affect Dox efficacy on primary tumor proliferation or apoptosis. At the end of the study, lung weight was recorded, and metastatic lung lesions were visualized by H&E (Figure 1G–J). Lung weight was significantly reduced in animals treated with Dox, antibiotics, and Dox + antibiotics compared to control animals (Figure 1H). However, only Dox + antibiotic-treated animals displayed a significantly reduced number of metastatic lung lesions, suggesting that antibiotics in combination with Dox reduced the metastatic potential (Figure 1I). This trend was also observed for the reduced metastatic lesion area in the Dox + antibiotics group (Figure 1J).

### 3.2. Doxorubicin Shifts the Gut Bacterial Microbiome

**Figure 2:**
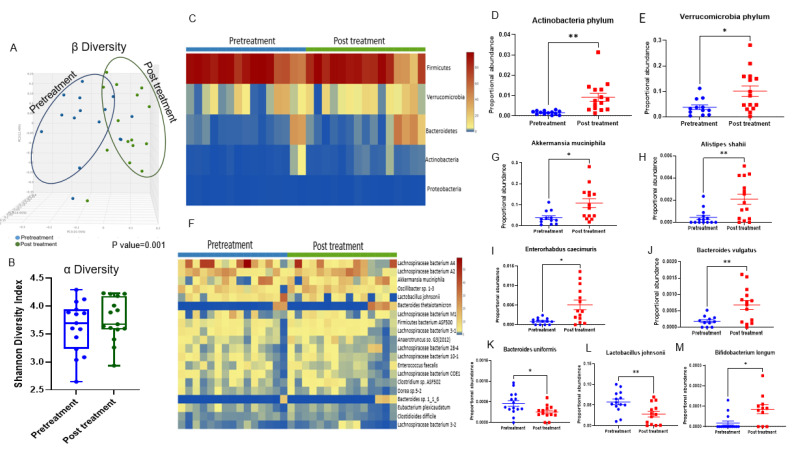
Metagenomics analysis of DNA from fecal samples that shows doxorubicin modulating the gut microbiota. (**A**) β-diversity principal coordinate analysis (PCoA) showing significantly different bacterial populations mediated by Dox administration, n = 15; *p* = 0.001. (**B**) Shannon index α-diversity measurement. n = 15. (**C**) Heatmap showing the proportional abundance of phyla before and after doxorubicin treatment. (**D**) Proportional abundance of *Actinobacteria* phylum before and after doxorubicin treatment. (**E**) Proportional abundance of *Verrucomicrobia* phylum. (**F**) Heatmap showing bacterial species modulated after doxorubicin treatment. (**G**) Proportional abundance of *Akkermansia muciniphilia*, (**H**) *Alistipes shahii*, (**I**) *Enterohabdus caecimuris*, (**J**) *Bacteroides vulgatus*, (**K**) *Bacteroides uniformis*, (**L**) *Lactobacillus johnsonii*, (**M**) *Bifidobacterium longum.* n = 11–15; * *p* < 0.05, **p < 0.005.

The analysis of fecal microbiota in the samples obtained from mice after treatment with oral antibiotics showed no significant differences in microorganisms detected compared to the negative control; therefore, data are not shown for this group. Fecal samples from mice treated with Dox at baseline (pre-treatment) and endpoint (after 4-weeks of Dox treatment) were analyzed to determine whether administration of Dox shifted the gut microbiome. As shown in (Figure 2A), Bray–Curtis β-diversity principal coordinate analysis (PCoA; comparing before and after treatment with doxorubicin) showed significant separation between the two communities (PERMANOVA, *p* = 0.001). However, there was no significant difference in α-diversity (Shannon diversity index) after Dox administration (Figure 2B). At the phylum level, Dox increased the proportional abundance of *Actinobacteria* and *Verrucomicrobia* (Figure 2C–E). At the species level, Dox treatment was associated with an increased proportional abundance of *Akkermansia muciniphila*, *Alistipes shahii*, *Bacteroides vulgatus*, *Enterorhabdus caecimuris (*Figure 2F–J), *Oscillibacter* sp. 1–3, *Oscillospiraceae bacterium* VE202-24, and *Prevotella copri* (Supplemental Figure S2B–D). Dox treatment was associated with a reduction in *Ruminococcus sp.* 5_1_39BFAA bacteria (Supplemental Figure S2A), reduced probiotic bacteria *Bacteroides uniformis* and *Lactobacillus johnsonii* (Figure 2K,L), and was associated with increased proportional abundance of *Bifidobacterium longum* (Figure 2M).

**Figure 3:**
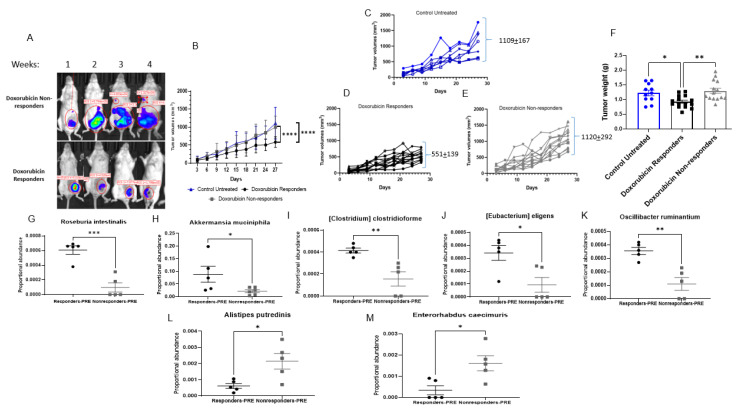
Doxorubicin response is associated with different baseline microbiome populations. (**A**) Representative images of In Vivo Imaging System (IVIS) of mice after each dose of doxorubicin injection over 4-week of treatment. (**B**) Tumor volumes of control untreated and doxorubicin (Dox) only treated group stratified into responder and nonresponder. n = 7–14, **** *p* < 0.0001. (**C**) Tumor volume of control untreated group in mm^3^, n = 7. (**D**) Tumor volume of Dox responder group in mm^3^, n = 14. (**E**). Tumor volume of Dox nonresponder group in mm^3^. n = 12. (**F**) Tumor weights in grams. * *p* = 0.03, ** *p* = 0.004. Proportional abundance of bacterial species before doxorubicin treatment between responder and nonresponder groups, (**G**) *Roseburia intestinalis*, (**H**) *Akkermansia muciniphila*, (**I**) *(Clostridium) clostridioforme*, (**J**) *(Eubacterium) eligens*, (**K**) *Oscillibacter ruminantium*, (**L**) *Alistipes putredinis*, (**M**) *Enterorhabdus caecimuris*. n = 5–6; * *p* < 0.01, ** *p* = 0.005, *** *p* = 0.0003.

Mice treated with Dox were stratified into responders and nonresponders based on the final tumor area (representative IVIS image, Figure 3A). Responders were classified from Dox-treated subjects that displayed a final tumor volume at least 2 standard deviations below the mean tumor volume of control animals. The mean tumor volume at the end of the study for Dox responders was 551 ± 139 mm^3^ vs. 1120 ± 292 mm^3^ for Dox nonresponders (Figure 3B–E). Responders displayed significantly reduced tumor weights compared to the Dox nonresponders (Figure 3F). Fecal bacterial microbiome populations were analyzed at baseline (prior to Dox administration), according to the treatment outcomes, to determine whether gut microbiome populations before treatment were associated with Dox response. Dox responders were associated with a higher abundance of *Roseburia intestinalis*, *Akkermansia muciniphila*, *(Clostridium) clostridioforme*, *(Eubacterium) eligens*, and *Oscillibacter ruminantium* (Figure 3G–K). At the same time, Dox nonresponders showed enrichment of *Alistipes putredinis* and *Enterorhabdus caecimuris* bacteria (Figure 3L,M). These findings were associated with changes in intra-tumoral macrophage recruitment (Supplementary Figure S1A), showing that Dox responders displayed higher intratumoral F4/80 immunoreactivity than Dox nonresponders, suggesting the importance of innate immune cell presence with better response to treatment. Altogether, these results indicate the importance of the gut microbiota as an indicator of Dox responsiveness.

### 3.3. Modulating the Gut Microbiome by High-Fat Diet Fecal Microbiota Transplantation (FMT) Reduces Dox Efficacy

**Figure 4:**
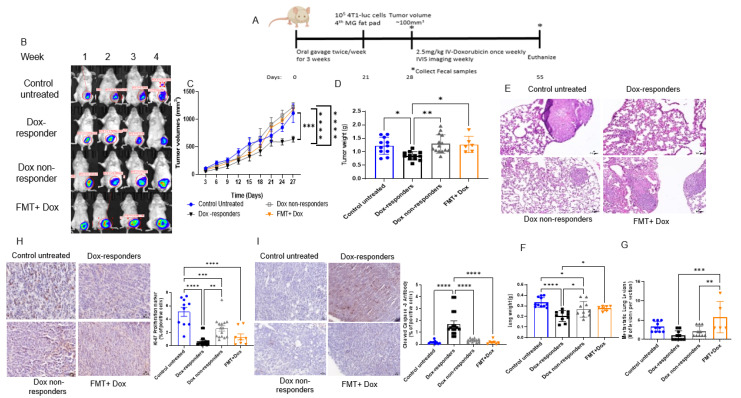
High-fat diet fecal microbiota transplantation (FMT) reduced tumor responsiveness to doxorubicin treatment. (**A**) Schematic design of murine TNBC model with high-fat diet FMT. Image produced by www.biorender.com. (**B**) Representative images of In Vivo Imaging System (IVIS) of mice after each dose of doxorubicin injection over 4-week of treatment. (**C**) Tumor volume was measured every three days and recorded in mm^3^. n = 6–14; *** *p* = 0.0001, **** *p* < 0.0001. (**D**) Tumor weight in grams. n = 6–14; * *p* < 0.02, ** *p* = 0.002. (**E**) Representative images of H&E-stained lungs from each treatment group. Lung weights (**F**), lung lesions (**G**). n = 5–11; * *p* < 0.05, ** *p* = 0.005, *** *p* = 0.0003, **** *p* < 0.0001. (**H**) Tumor proliferation marker Ki-67 by IHC immunoreactivity. n = 8–15; ** *p* = 0.001, *** *p* = 0.0005, **** *p* < 0.0001. (**I**) Apoptosis marker (cleaved caspase 3) in tumors by IHC immunoreactivity. n = 5–15; **** *p* < 0.0001.

Elevated visceral adiposity is associated with reduced disease-free survival in neoadjuvant chemotherapy outcomes in advanced breast cancer patients, suggesting that adiposity modulates chemotherapy responsiveness [36]. Next, we investigated the effect of the high-fat diet microbiome on Dox responsiveness (Figure 4A). High-fat FMT in the mice that consumed a low-fat control diet resulted in reduced chemotherapy responsiveness, as determined by a significant increase in tumor volume and weight compared with Dox responders (Figure 4B–D). Lung weight and lung metastatic lesion number were significantly higher in FMT + Dox-treated mice than in Dox responders (Figure 4E–G). While tumors from FMT + Dox-treated mice showed reduced proliferation when compared with control mice (as determined by Ki67 immunoreactivity (Figure 4H)), tumors from FMT + Dox-treated animals showed a significant reduction in cleaved caspase 3 when compared with Dox responders (Figure 4I), indicating that high-fat diet FMT prevented Dox-mediated apoptosis from affecting efficacy. These findings were associated with lower intra-tumoral macrophage recruitment (Supplementary Figure S1B), showing a significant reduction of the F4/80 marker in tumors of FMT + Dox compared to the Dox responder group. Furthermore, metagenomic sequencing confirmed the efficacy of FMT in shifting gut microbiota (Supplementary Figure S3). A high-fat diet FMT increases the abundance of many bacterial species, including *Bacteroides facies*, *Bacteroides thetaiotaomicron*, *Bacteroides vulgatus*, *Enterorhabdus caecimuris*, *Parabacteroides distasonis*, *Lachnospiraceae bacterium* 28–4 and *Parabacteroides* sp. D13 (Supplemental Figure S3A–H). Of particular interest, high-fat diet FMT resulted in a 2-fold increase in the gut proportional abundance of *Enterorhabdus caecimuris*, a microbe associated with Dox administration (Figure 2I) and, more importantly, with the Dox nonresponder phenotype (Figure 3M). These findings suggest that the poor clinical outcomes observed in obese patients with breast cancer treated with chemotherapy may be mediated, in part, by the gut microbiome.

### 3.4. Shifting Gut Microbiota through Chemotherapeutic Treatment and HFD-FMT Increased Plasma LPS Levels and Caused Intestinal Permeability

**Figure 5:**
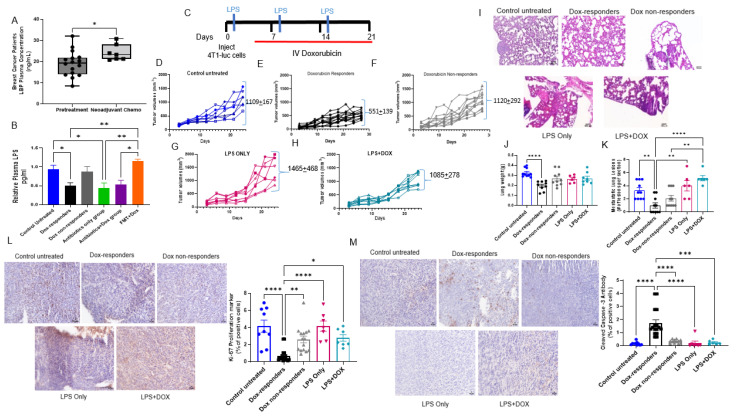
Exogenous LPS affected chemotherapy responsiveness. (**A**) ELISA (enzyme-linked immunosorbent assay) on human plasma samples measuring LPS-binding protein (LBP). n = 7–16; * *p* = 0.02, (**B**) ELISA on mice plasma sample measuring circulating LPS. n = 10–15; * *p* = 0.01, ** *p* < 0.005. (**C**) Schematic design of murine TNBC model with exogenous LPS injection. (**D**–**H**) Tumor volume of study groups was measured every three days and recorded in mm^3^**,** control untreated (**D**), Dox responders (**E**), Dox nonresponders (**F**), LPS only (**G**), and LPS + DOX (**H**), n = 7–12. (**I**) Representative images of H&E-stained lungs from each treatment group. Lung weights (**J**), and lung lesions (**K**), measured in the lungs. n = 5–10; ** *p* < 0.005, **** *p* < 0.0001. (**L**) Tumor proliferation marker Ki-67 in tumors measured by IHC immunoreactivity. n = 6–15; * *p* = 0.01, ** *p* = 0.002, **** *p* < 0.0001. (**M**) Apoptosis marker (cleaved caspase 3) in tumors measured by IHC immunoreactivity. n = 6–15; *** *p* < 0.0005, **** *p* < 0.0001.

Elevated plasma LPS levels indicate leaky gut and metabolic endotoxemia [37]. Obesity elevates metabolic endotoxemia, and LPS bioavailability may represent a potential molecular mechanism that mediates Dox response [38]. We measured the LPS binding protein (LBP) concentration in plasma samples obtained from breast cancer patients, either before systemic adjuvant treatment or after receiving neoadjuvant chemotherapy. Patient demographic data are presented in (Supplementary Table S1). Circulating LBP levels were elevated in the plasma of patients treated with neoadjuvant chemotherapy compared to the plasma taken from patients before systemic therapy (Figure 5A). We also measured plasma samples of mice treated with Dox, antibiotics, and FMT to determine circulating LPS concentrations. The Dox nonresponders and FMT + Dox groups showed an increase in circulating LPS levels compared with Dox responders (Figure 5B), suggesting elevated intestinal inflammation and gut permeability in these groups. To further explore the impact of LPS bioavailability on Dox efficacy, we co-treated 4T1-bearing mice with LPS and Dox. (Figure 5C). There were no differences in end tumor volume between the control untreated, Dox nonresponders, LPS, or LPS + Dox-treated mice, suggesting that elevated LPS bioavailability reduces Dox efficacy (Figure 5D–H). In addition, there was no difference in lung weights between LPS, LPS + DOX and control untreated groups; however, LPS and LPS + DOX-treated groups showed an increase in lung metastatic lesions compared to Dox responders (Figure 5I–K). The LPS-treated groups showed a significant increase in tumor proliferation compared to the Dox responders (Figure 5L). In addition, we observed a significant reduction in cleaved caspase 3 immunoreactivity in tumors from LPS-treated groups compared to the Dox responder group (Figure 5M), suggesting that LPS signaling reduces Dox-mediated apoptotic signaling to reduce chemotherapy anti-cancer response. These findings were associated with an abrogated intratumoral F4/80 macrophage population compared to the Dox responders (Supplemental Figure S1C). Taken together, these data suggest that elevated LPS bioavailability was associated with a decrease in the efficacy of chemotherapy for TNBC.

### 3.5. Modulating Gut Microbiota Affected Intestinal Health and Increased Intestinal Inflammation

**Figure 6:**
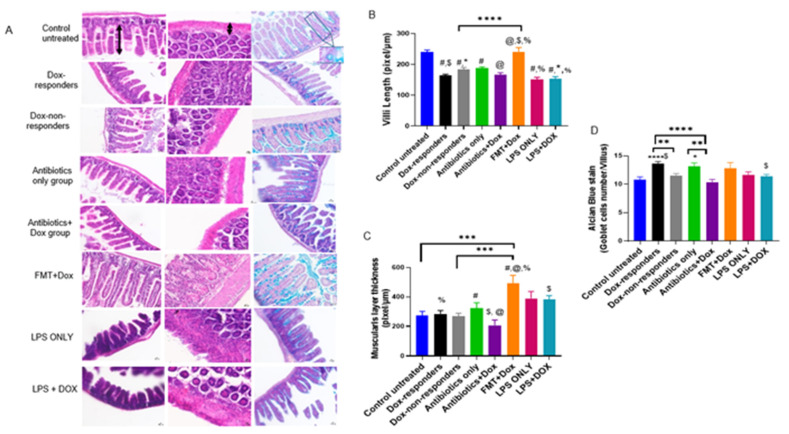
Intestinal inflammation parameters in TNBC murine model with different interventions. (**A**) Representative images of H&E-stained and Alcian blue-stained intestines from each treatment group, showing intestinal inflammation parameters and goblet cell staining. (**B**) Villi length measured in pixel/µm in the small intestine. N = 6–10; * *p* < 0.02, ****^,$,#,@,%^ *p* < 0.0001. (**C**) Muscularis thickness measured in pixel/µm in the large intestine. N = 6–10; * *p* = 0.02, ^%^ *p* = 0.002, *** *p* < 0.0009, ^$^ *p* = 0.03, ^#^ *p* = 0.02, ^@^ *p* = 0.0002. (**D**) Alcian blue staining for small intestines to stain goblet cells on villi. N = 6–10; * *p* = 0.01, **** *p* < 0.000, ^$,^** *p* < 0.004.

The intestinal epithelium is considered one of the most rapidly proliferating tissues in the body, making the gut sensitive to chemotherapeutic drug side effects [39]. In this study, intestinal tissues were stained with H&E and Alcian blue to show the intestinal damage features caused by therapy and interventions. We measured villi length, muscularis thickness, and goblet cell counts. Most groups showed a significant reduction in villus length compared to the untreated control group, except for the high-fat diet FMT + Dox group (Figure 6A,B). Dox responders and Dox + antibiotic-treated groups showed significantly shorter villi than the FMT + Dox group. LPS-treated groups showed shorter villi than the FMT + Dox group. Muscularis thickness findings showed a significant increase in the FMT + Dox group compared with all other study groups (Figure 6A,C). The LPS + Dox group showed an increase in muscularis thickness compared to the Dox + antibiotic-treated group. Dox responders showed a significant increase in goblet cell count per villus; however, the Dox + antibiotic-treated group showed a significant reduction in goblet cells compared to the Dox responders and antibiotic-only treated groups. The LPS + Dox group showed a significant reduction in goblet cell number compared to Dox responders (Figure 6A,D). Altogether, these findings emphasize the effect of systemic intervention in modulating gut microbiota content related to changes in gut epithelium and intestinal inflammation, which may lead to increased gut permeability associated with the modulation of tumor responsiveness to treatment.

## 4. Discussion

The microbiome is important for human body development. Changes in the composition and distribution of the gut microbiome can cause many health problems, including obesity, inflammatory bowel disease, and cancer [40]. Anti-cancer therapies can act as a selection pressure to shift the gut and tumor microbiota [30,41]. These agents may also lead to toxic side effects, such as neutropenia or mucositis. These side effects are often treated with antibiotics that may further promote gut dysbiosis by reducing the abundance of healthy gut microbes and impacting the biological processes associated with these microbiotas, which may result in a defect in immune system development and activities [42]. Several studies have demonstrated the role of antibiotics in cancer outcomes, either by promoting anti-tumor effects or compromising treatment efficacy [43]. For example, cyclophosphamide (CTX) can kill cancerous cells through many mechanisms, including inducing immunogenic cancer cell death, subverting immunosuppressive T cells, and promoting Th1 and Th17 responses to control cancer growth [44]. After sterilization of the gut by broad-spectrum antibiotics, there was an observed reduction in IL-17 and IFN-ɤ producing T cell populations, reducing the anti-cancer effect of CTX on subcutaneous cancer-bearing mice [21]. Our study focused on doxorubicin chemotherapy, an anthracycline that displays anti-tumor effects by producing free radicals that cause lipid peroxidation, DNA intercalation, and inhibiting topoisomerase II enzyme, thereby preventing DNA replication and causing cell death [8]. Herein, we demonstrate the effectiveness of broad-spectrum antibiotics (subsequently ablating the gut microbiota) during treatment with Dox chemotherapy. This broad-spectrum antibiotic cocktail has previously been shown to be effective in depleting the gut microbiome [21]. We found that antibiotics combined with doxorubicin improved the outcomes in the 4T1 TNBC murine model, which was associated with reduced tumor proliferation and elevated apoptosis. The combination of Dox+ antibiotic administration also reduced the number of breast cancer lung metastatic lesions, suggesting that gut microbiota and/or tumor microbiota may contain bacteria that not only promote tumor growth but also enhance migration and metastasis development. Interestingly, it was recently shown in a murine spontaneous breast tumor model (MMTV-PyMT) that intracellular microbiota could travel through the circulation with cancer cells and play a crucial role in metastasis by modulating the cellular cytoskeleton and cell viability without affecting primary tumor growth [31]. Further findings from the metagenomic analysis of fecal samples showed that Dox administration shifted the gut microbiota distribution and community from baseline. At the species level, Dox treatment was associated with increased *Akkermansia muciniphila*, *Alistipes shahii*, *Prevotella copri*, *Bacteroides vulgatus*, *Enterorhabdus caecimuris*. Doxorubicin was associated with a reduction in some probiotic bacteria proportional abundance, such as *Bacteroides uniformis* and *Lactobacillus johnsonii,* but was associated with an increase in *Bifidobacterium longum*. These findings emphasize doxorubicin’s role in shifting gut microbiota contents.

A previous study showed that *A. muciniphila* was reduced in the gut of women who developed breast cancer during their lifetime [45]. In addition, in line with the association with breast cancer, type 2 diabetes, and high BMI are associated with a reduced proportional abundance of *A. muciniphila* [46], suggesting that an increase in *A. muciniphila* may be beneficial. When comparing doxorubicin responders and nonresponders, an elevated proportional abundance of *A. muciniphila* was observed in Dox responder pretreatment, highlighting that this microbe may be a biomarker of Dox response and that enriching the gut with this bacterium may improve Dox responsiveness in TNBC patients. Supplementation of *A. muciniphila* in overweight and obese volunteers showed that daily administration of 10^10^ CFU of pasteurized or live bacteria for 3 months resulted in increased gut *A*. *muciniphila* abundance and improved insulin sensitivity parameters. Supplementation was well tolerated, and the clinical trial (NCT02637115) demonstrated that *A. muciniphila* supplementation is safe [47]. In a preclinical model, C57BL/6 mice bearing E0771 tumors fed a high-fat diet and treated with immune checkpoint blockade (anti-PD-1) therapy showed that elevated gut *A. muciniphila* in obese mice after treatment with anti-PD-1 was associated with favorable outcomes [48]. Clinically, TNBC patients eligible for immune checkpoint blockade are treated with a combination of anti-PD-1 antibodies and chemotherapy; therefore, our preclinical data suggest that a Dox-associated increase in gut *A. muciniphila* may promote immune checkpoint therapy responsiveness. *A. muciniphila* could impact therapy responsiveness through several mechanisms; *A. muciniphila* is a mucin-degrading bacteria and produces short-chain fatty acids, which can increase mucin production and further protect the gut epithelium [49]. *A. muciniphila* can reduce inflammatory process activation in the gut by reducing inflammatory cytokines, such as TNF-α, IL-6, and IL-12 [50]. However, further studies are needed to explore *A. muciniphila’s* molecular mechanisms in promoting chemotherapy and immunotherapy responsiveness in TNBC.

A previous study showed that *Prevotella copri* bacteria are more common in plant (fiber)-rich diets and are normally highly abundant in healthy human gut microbiota [51]. *P. copri* has a dual role, depending on the diet consumption of the host; this bacterium is associated with diseases such as hypertension and diabetes in the Westernized population [52]. In our study, we showed that *P. copri* increased after doxorubicin treatment but was not associated with responsiveness. A non-human primate study showed that the subjects who consumed a Westernized diet with kidney dysfunction markers displayed elevated gut *P. copri* proportional abundance, suggesting that this microbe modified by Dox administration may promote chemotherapy toxicities, such as kidney damage [33]. Other reports have highlighted that *B. uniformis* supplementation reduces metabolic dysfunction and inflammation in mice fed a high-fat diet [53]; Inflammation can be a critical mediator of Dox efficacy [54]. The current study reports found that Dox treatment reduced the proportional abundance of *B. uniformis,* potentially linking this microbe to therapy-induced inflammation. Further studies are needed to determine the role of gut *B. uniformis* on Dox efficacy.

The *Enterorhabdus* genus is a member of the *Actinobacteria* phylum associated with ileocecal mucosal inflammation [55]. *Enterorhabdus caecimuris* was isolated from the ileocecal regions of mice suffering from colitis and intestinal inflammation [56]. We now show that the proportional abundance of this bacterium is elevated after Dox treatment and is associated at baseline with Dox nonresponders, suggesting that this microbe may play a role in intestinal inflammation to promote gut permeability and/or bacterial translocation, which may enhance metastatic potential and counteract cancer cell killing effects mediated by chemotherapy. Our study suggests that the gut microbiota abundance of *A. muciniphila* (responders) or *E. caecimuris* (nonresponders) species prior to treatment with chemotherapy could be used as a biomarker of responsiveness to treatment.

Emphasizing the critical role of gut microbiota content prior to chemotherapy, we focused on modulating gut microbiota with high-fat diet-derived (HFD)-FMT as a non-dietary method to modulate the gastrointestinal microbiota to show the causality of the gut microbiome in regulating chemotherapy response. We previously showed that consumption of a high-fat diet or HFD-FMT in female mice shifted the gut microbiome and increased breast cancer risk [29]. In the current study, HFD-FMT reduced Dox responsiveness and increased the development of distant metastasis. We showed that HFD-FMT reduced the Dox response by preventing chemotherapy-induced apoptosis. At the intestinal inflammation level, HFD-FMT promoted intestinal inflammation, as marked by increased muscularis thickness and reduced goblet cell count when treated with Dox. These findings highlight the role of shifting gut microbiota on intestinal health and chemotherapy efficacy.

A previous study carried out by our group showed elevated circulating plasma LPS in mice fed a high-fat diet and in mice fed a control diet that was administered with HFD-FMT, suggesting that increased gut permeability and metabolic endotoxemia could be mediated by high-fat diet-regulated microbes [29]. Our current study showed that breast cancer patients who received neoadjuvant chemotherapy showed higher plasma levels of lipopolysaccharide-binding protein as an indicator of circulating proinflammatory LPS levels compared to treatment-naïve patients. In addition, circulating LPS was elevated in Dox nonresponder mice and the FMT + Dox group, suggesting that LPS bioavailability is a potential mediator of chemotherapy efficacy in our preclinical models. In a previous study, intraperitoneal LPS administration in a TNBC murine model increased the number and size of lung metastatic lesions associated with elevated angiogenesis [32]. We showed that administration of exogenous LPS decreased the Dox response and increased lung metastasis formation, similar to HFD-FMT administration. LPS is a structural component of Gram-negative bacteria and can bind to toll-like receptor 4 (TLR4), activating several signaling mechanisms that may affect cell survival, including proliferation, inflammation, and apoptosis [57]. Overall, our data suggest that LPS can increase tumor proliferation and disrupt Dox apoptotic capacity and reduce tumor response to treatment.

Doxorubicin is known for causing mucositis as a side effect; Dox causes transient mucosal damage to the jejunum, including decreased crypt proliferation, crypt number, and villus height [58]. Gut microbiota is crucial for the initiation and maintenance of mucosal damage and repair; however, the role of the gut microbiota in regulating Dox-mediated intestinal damage is underexplored. A potential mechanism of Dox action on the gut epithelium could be explained by increased intestinal epithelial barrier permeability to small proinflammatory molecules, such as lipid A and/or LPS, resulting in immune system activation and endotoxemia [59]. Endotoxins bind to TLR receptors and promote systemic inflammation. In addition, Dox administration increases the expression of TLR4 in macrophages, leading to increased inflammation and damage in several organs, suggesting that Dox regulation of LPS-containing microbes, gut leakiness, and regulation of TLR receptors on immune cells represents a multifactorial mechanism by which chemotherapy can regulate inflammation [60]. These findings may emphasize the role of gut microbiota in exaggerating the damage from Dox treatment, and modulating gut microbiota may greatly reduce severe side effects and toxicity.

A recent study highlighted the potential role of chemotherapy in the modulation of the oral microbiome in association with the development of oral side effects. This study characterized the effect of chemotherapeutic drugs on the oral microbiome through 16S-rRNA sequencing of saliva samples from 20 breast cancer patients before and after treatment sessions, demonstrating that chemotherapy was associated with a significant increase in the relative abundance of potentially pathogenic taxa, such as Escherichia/Shigella, emphasizing that oral microbiota could be used as a potential target to treat common oral side effects during cancer patients therapy [61].

The literature has shown that dysbiosis can promote carcinogenesis through several mechanisms, including unregulated inflammatory processes induced by bacterial metabolites [62], microbiome-mediated immune programming [63], and gut barrier dysfunction [64]. These data suggest that intact intestinal barrier components are crucial in the carcinogenesis cascade by preventing bacterial translocation from the gut into distant organs or modulating LPS leakage into circulation, thereby controlling systemic inflammation. Moreover, preclinical studies are emerging, indicating how one can shift gut microbiome populations through exercise and that prebiotic fiber intake to result in improved breast cancer outcomes [65], suggesting that the gut microbiome is plastic, modifiable, and targetable risk factor for breast cancer.

## 5. Conclusions

In conclusion, the interaction between gut microbiota, gut integrity, and the immune system may provide several novel targets to improve cancer therapy responsiveness and TNBC survival. In this study, we showed that ablation of the gut microbiome through antibiotic administration promoted Dox chemotherapy efficacy, suggesting that certain microbes decrease the effectiveness of chemotherapy and promote metastasis. In addition, we highlighted the importance of gut microbiota in modulating chemotherapy responsiveness in treating TNBC and the potential for the gut microbiome to be used as a biomarker for chemotherapy responsiveness. We identified *A. muciniphila* as a potential gut microbe that promotes Dox response and gut *E. caecimuris* as a microbe associated with reduced therapeutic efficacy. Indeed, shifting the gut microbiota using a high-fat diet FMT method that increased gut *E. caecimuris* showed a reduced response to Dox. Finally, we demonstrated the role of the bacterial endotoxin LPS in reducing Dox responsiveness, suggesting that increasing gut permeability promotes drug efficacy and metastases.

However, the use of antibiotics in cancer patients and the safety of FMT remain controversial, and the quality of evidence for modulating the gut microbiota in cancer management generally remains low. Herein, we showed that the gut microbiota could be a double-edged sword; microbiota could have a pro-carcinogenic effect or may increase the response to chemotherapy treatment. We further emphasized the crosstalk between the gut and tumor, where gut integrity and microbiota metabolites play vital roles in shaping the outcomes of neoadjuvant chemotherapy treatment of breast cancer patients.

## Data Availability

The data presented in this study are available in this article (and supplementary material).

## Supplementary Materials

The following supporting information can be downloaded at: https://www.mdpi.com/article/10.3390/cancers14194849/s1, Figure S1: Macrophage recruitment into tumors; Figure S2: Proportional abundance of bacterial species modified post doxorubicin treatment; Figure S3: Metagenomic analysis of fecal samples showing the proportional abundance of bacterial species modified after high-fat diet derived-FMT; Table S1: Patients’ breast cancer characteristics. Data are presented as mean ± standard deviation.
