# Supplementary material for: Intestinal Microbiota Influence Doxorubicin Responsiveness in Triple-Negative Breast Cancer
Source: Cancers (Basel). 2022 Oct 4;14(19):4849. doi: 10.3390/cancers14194849 (PMC9563306; doi:10.3390/cancers14194849)
Supplement: Supplementary file 1 [file cancers-14-04849-s001.zip › cancers-1884154-supplementary.pdf]

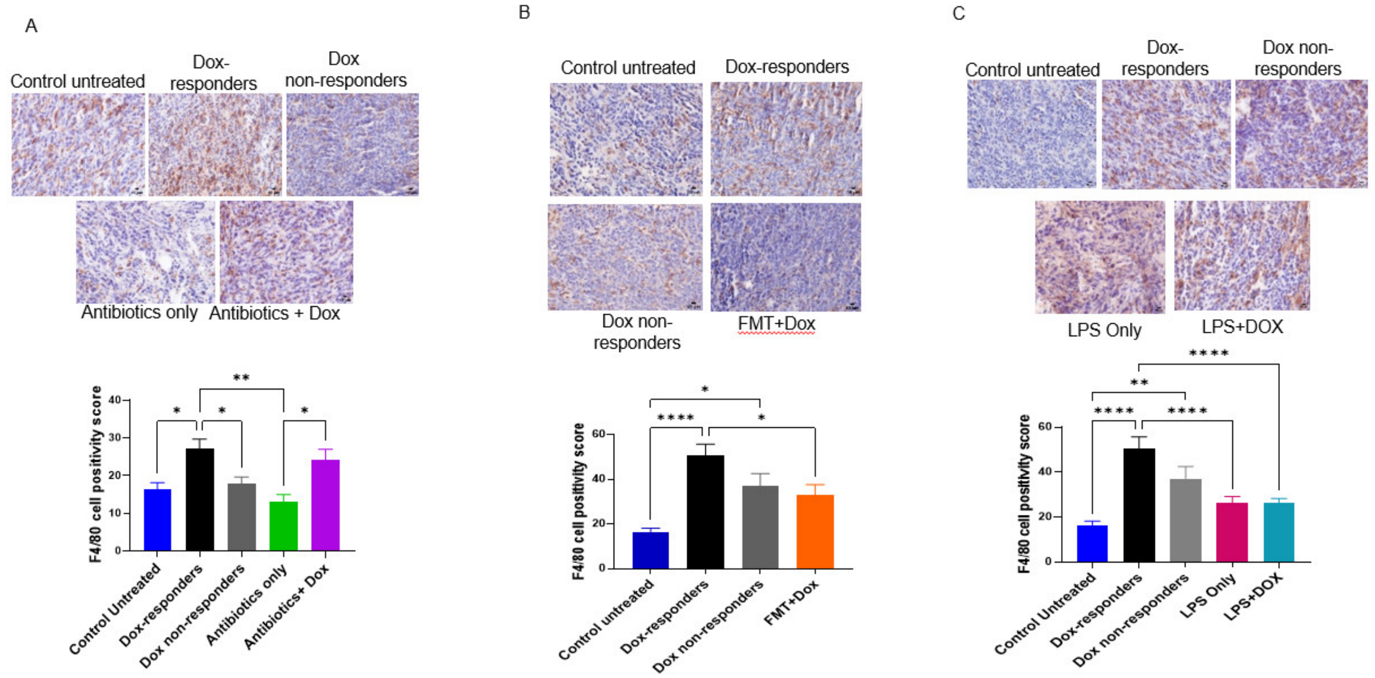

Supplementary Figure S1: Macrophage recruitment into tumors. **A.** Macrophage marker F4/80 shown in IHC images of tumors in a murine TNBC model. n=10; *p=0.05, **p=0.001. **B.** Macrophage marker F4/80 shown in IHC-stained tumors in a high-fat diet derived-FMT study TNBC murine model. n=5-10; *p=0.01, ****p<0.0001. **C.** Macrophage marker F4/80 shown by IHC in an inflammatory TNBC murine model. n=7-10; **p=0.002, ****p<0.0001.

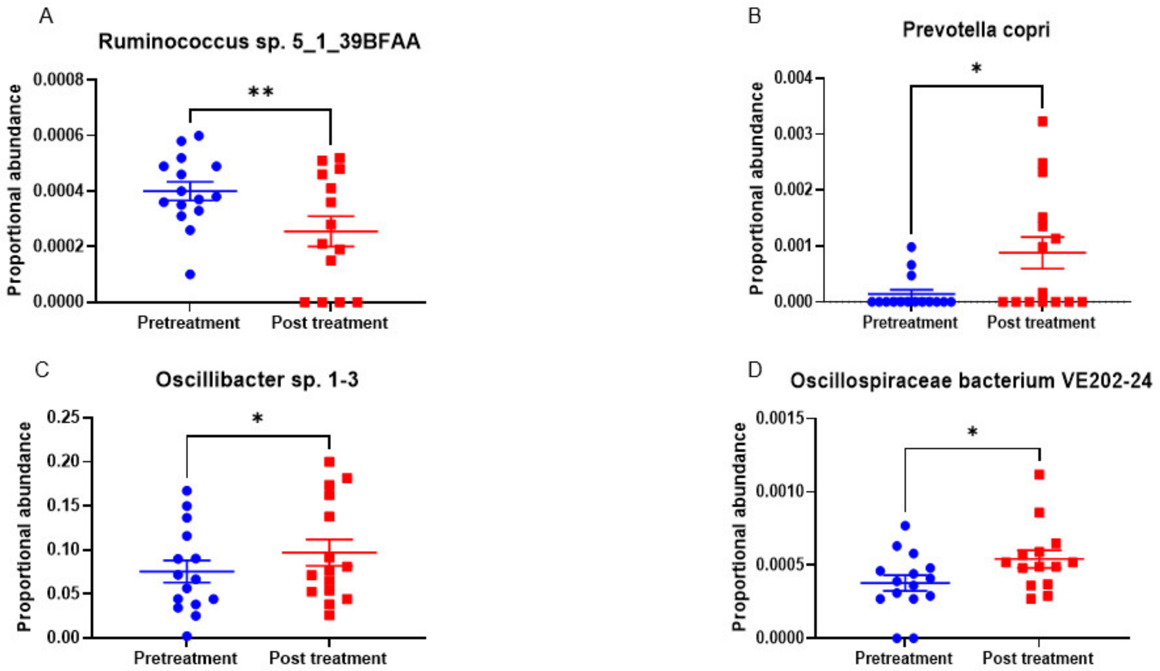

Supplementary Figure S2: Proportional abundance of bacterial species modified post doxorubicin treatment. A. *Ruminococcus sp.5_1_39BFAA* bacterial species were reduced after Doxorubicin treatment. n=15; **p=0.005. **B.** Proportional abundance of bacterial species that increased after doxorubicin treatment, *Prevotella copri***,** **C.** *Oscillibacter sp. 1-3*, **D.** *Oscillospiraceae bacterium VE202-24*. n=15; *p=0.05.

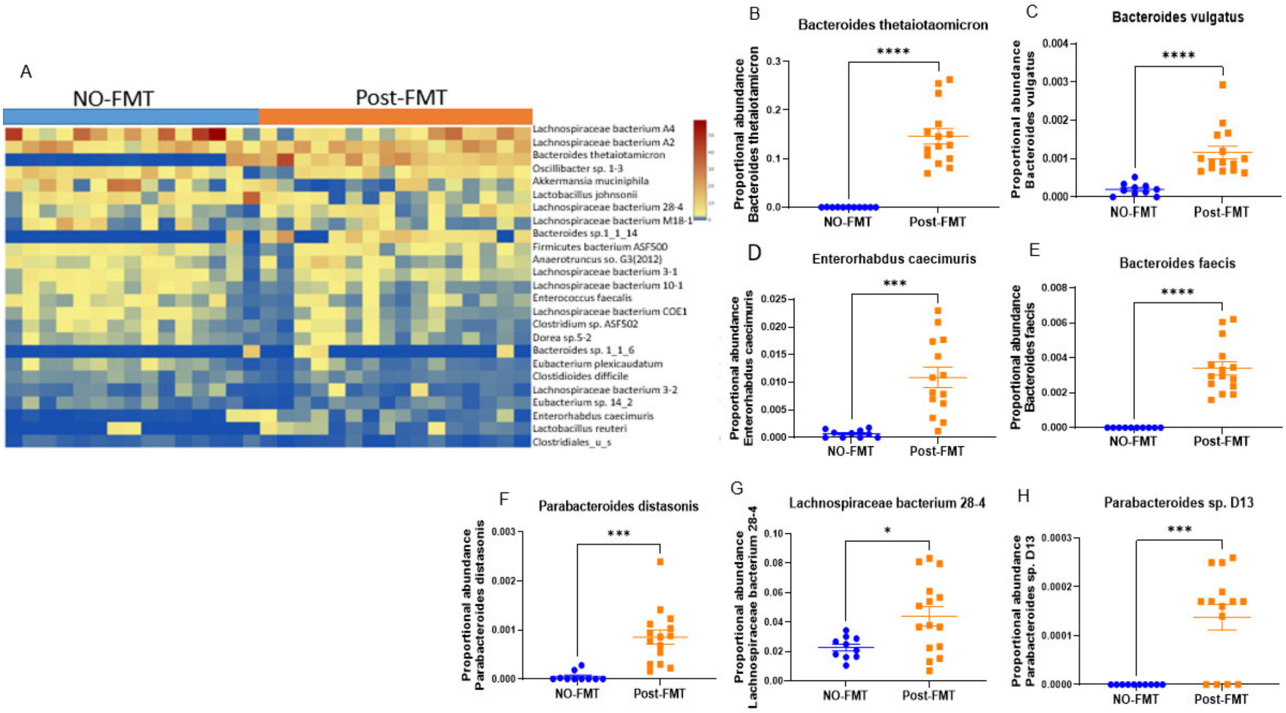

Supplementary Figure S3: Metagenomic analysis of fecal samples showing proportional abundance of bacterial species modified after high fat diet derived-FMT. A: Heatmap of the study groups at baseline (No-FMT) and endpoint (post-FMT). Bacterial species that modulated and increased abundance post FMT **B.** *Bacteroides thetaiotaomicron,* **C.** *Bacteroides vulgatus,* **D.** *Enterohabdus caecimuris,* **E.** *Bacteroides faecis,* **F.** *Parabacteroides distasonis,* **G.** *Lachnospiraceae bacterium 28-4,* **H.** *Parabacteroides sp. D13.* n=10-15; *p=0.01, ***p=0.0001, ****p<0.0001.

Supplementary Table S1: Patients' breast cancer characteristics. Data are presented as mean ± standard deviation.

	Pretreatment group, n=16	Neoadjuvant chemotherapy group, n=7
Age, years	60.5±11.2<br>(38-81)	61.6±11.5<br>(48-77)
BMI, kg/m^2	33.1±7.4<br>(25.8-56.6)	35.3±10.2<br>(27.1-57.4)
Ethnicity<br>Caucasian<br>African American	 75%<br>25%	 86%<br>14%
Tumor classification<br>Ductal	 100%	 100%
Tumor receptor status<br>ER+/PR+<br>HER2+<br>TNBC	 12.5%<br>25%<br>62.5%	 0%<br>43%<br>57%
